# In Vitro Study of a Novel *Vibrio alginolyticus*-Based Collagenase for Future Medical Application

**DOI:** 10.3390/cells12162025

**Published:** 2023-08-08

**Authors:** Lindsey Alejandra Quintero Sierra, Reetuparna Biswas, Alice Busato, Anita Conti, Riccardo Ossanna, Giamaica Conti, Nicola Zingaretti, Michele Caputo, Christian Cuppari, Pier Camillo Parodi, Andrea Sbarbati, Michele Riccio, Francesco De Francesco

**Affiliations:** 1 Department of Neuroscience, Biomedicine, and Movement Sciences, Human Anatomy and Histology Section, University of Verona, 37134 Verona, Italy; lindseyalejandra.quinterosierra@univr.it (L.A.Q.S.); reetuparna.biswas@univr.it (R.B.); busatoalice@gmail.com (A.B.); anita.conti@univr.it (A.C.); riccardo.ossanna@univr.it (R.O.); giamaica.conti@univr.it (G.C.); andrea.sbarbati@univr.it (A.S.); 2 Clinic of Plastic and Reconstructive Surgery, Academic Hospital of Udine, Department of Medical Area (DAME), University of Udine, 33100 Udine, Italy; zingarettin@gmail.com (N.Z.); piercamillo.parodi@uniud.it (P.C.P.); 3 Fidia Farmaceutici S.p.A., R&D Local Unit Fidia Research Sud, Contrada Pizzuta, 96017 Noto, Italyccuppari@fidiapharma.it (C.C.); 4 Research and Training Center in Regenerative Surgery, Accademia del Lipofilling, 61025 Montelabbate (PU), Italy; 5 Department of Reconstructive Surgery and Hand Surgery, AOU “Ospedali Riuniti”, 60126 Ancona, Italy

**Keywords:** collagenase, *Vibrio alginolyticus*, regenerative medicine, adipose tissue, disaggregation

## Abstract

Mesenchymal stem cells extracted from adipose tissue are particularly promising given the ease of harvest by standard liposuction and reduced donor site morbidity. This study proposes a novel enzymatic method for isolating stem cells using *Vibrio alginolyticus* collagenase, obtaining a high-quality product in a reduced time. Initially, the enzyme concentration and incubation time were studied by comparing cellular yield, proliferation, and clonogenic capacities. The optimized protocol was phenotypically characterized, and its ability to differentiate in the mesodermal lineages was evaluated. Subsequently, that protocol was compared with two *Clostridium histolyticum*-based collagenases, and other tests for cellular integrity were performed to evaluate the enzyme’s effect on expanded cells. The best results showed that using a concentration of 3.6 mg/mL *Vibrio alginolyticus* collagenase allows extracting stem cells from adipose tissue after 20 min of enzymatic reaction like those obtained with *Clostridium histolyticum*-based collagenases after 45 min. Moreover, the extracted cells with *Vibrio alginolyticus* collagenase presented the phenotypic characteristics of stem cells that remain after culture conditions. Finally, it was seen that *Vibrio alginolyticus* collagenase does not reduce the vitality of expanded cells as *Clostridium histolyticum*-based collagenase does. These findings suggest that *Vibrio alginolyticus* collagenase has great potential in regenerative medicine, given its degradation selectivity by protecting vital structures for tissue restructuration.

## 1. Introduction

Regenerative cell therapy, which passes on the therapeutic accomplishment of stem cells to recover diseased or damaged tissue, has obtained increasing consideration from scientists and clinicians [1,2]. Mesenchymal Stem Cells (MSCs) are multipotent fibroblast-like cells capable of self-renewal and differentiate under adequate stimuli into different cell lineages such as adipocytes, chondrocytes, or osteoblasts that can be harvested from diverse adult tissues [3,4,5,6]. MSCs are responsible for maintaining the functionality of the body by substituting cells that are no longer able to accomplish their role in an organ or tissue [7]. It has been found that MSCs not only present the capacity to differentiate into a variety of cell types but also possess the ability to secrete high quantities of cytokines and growth factors (fibroblast growth factor, keratinocyte growth factor, IL-6, and IL-7, among others), increasing the MSCs’ effect in the reparative processes [8]. MSCs carry considerable engagement for tissue regeneration due to their essential capability to supply a renewable contribution of progenitor cells that can build several cell varieties, whole tissue structures, and organs [9,10]. The primary sources used to isolate MSCs are some adult and fetal tissues such as amniotic fluid, peripheral blood, bone marrow, adipose tissue, and so forth [11]. Among these, adipose tissue stands out the most as it is an abundant and available supply of MSCs and is considered as discarded tissue after procedures of reconstructive and plastic surgery [12]. For this reason, autologous adipose-derived stem cells (ASCs) have developed into highly interesting targets for use in regenerative cell therapy, given their high availability and capability to differentiate into different cellular lineages [13,14,15,16,17,18]. The clinical use of ASCs is increasing rapidly because of their encouraging results across a wide range of clinical applications [19,20,21].

Optimizing the isolation of ASCs is important not only to improve the efficiency of the extraction but also for the correct identification of the physiological level of extracted ASCs, which determines their possible clinical applications [22]. Different ASC isolation methodologies have been studied to optimize the extraction process, separating the highest feasible amount of living ASCs from the lowest achievable quantity of adipose tissue in the shortest workable time. Some of these methods are focused on mechanical isolation using shear force, centrifugal force, or turbulence force [23,24,25,26,27,28,29,30,31,32], and some others use enzymatic digestion [33,34]. In this last regard, bacterial collagenase is the most conventional proteolytic enzyme used for the disaggregation of tissues [35]. The most popular commercially available bacterial collagenase is a lyophilized extract of the anaerobic culture of *Clostridium histolyticum*. Among the best-known uses of *C. histolyticum* collagenase in the medical field are wound debridement [36,37], treatment of Dupuytren’s disease [38,39,40,41], and treatment of collagen plaque in the tunica albuginea of Peyronie’s disease [42,43]. However, *C. histolyticum* collagenases present low collagen selectivity, degrading other membrane proteins such as fibronectin and decorin, which are fundamental components of the extracellular matrix [44]. The low selectivity of *C. histolyticum* collagenases is probably due to the variability in their proteolytic composition [45]. In this regard, the study on collagenases derived from diverse bacteria strains has gained attention. Those of the genus *Vibrio* are one of the most encouraging bacteria, a non-pathogenic strain well known as protease producers and worthy of more attention and investigation as a wellspring of enzymes [46,47]. The first reported collagenolytic enzyme was the collagenase from *Vibrio alginolyticus*, a chemovar *iophagus* bacteria which was first named *Achromobacter collagenase* or *achromase* but recently known as *Vibrio collagenase*. The *Vibrio alginolyticus*-based collagenase is a highly purified single-band protein (molecular weight 81,875 Da) that does not contain non-specific proteases or other microbial impurities. *V. alginolyticus*-based collagenase is an extracellular metalloproteinase with a specific activity towards collagen substrates [44,46,48]; its amino acid sequence does not bear significant similarity to other collagenases [49]. Due to its low metabolic activity against fibronectin and decorin, the primary utility of *Vibrio collagenases* depends on their capability to perform the careful eradication of necrotic tissue [44,50] with only minor damage to the periwound healthy tissues [51,52]. *V. collagenase* has been established to be valuable and adequate as a debriding instrument and is being recommended for pharmaceutical use [53]. The value and accordance of collagenase have been a recurring theme in the literature between batches and manufacturers [54]. Moreover, at the present day, the use of enzymes implies high costs and might impact safety and efficacy [55,56].

The translation of research-based methods into a process for the large-scale preparation of clinical-grade ASCs according to the Good Manufacturing Practice rules is crucial and firmly based on the safety, purity, and efficacy of the cells. The procedure demands rigorous quality control validation at all pivotal points during the fabrication process [57].

This study aimed to evaluate a novel *Vibrio* collagenase enzyme for the isolation of adipose-derived stem cells, comparing cellular yield, cellular viability, and number of living cells per ml of lipoaspirate with the standard *Clostridium* collagenase used in research.

## 2. Materials and Methods

### 2.1. Adipose Tissue Collection

The adipose samples were collected from 30 patients after obtaining informed consent for a liposuction procedure. Sample collection followed the ethical guidelines established by the review committee for human studies. To obtain the adipose sample, the patient was injected with Klein tumescence solution (2% lidocaine solution: 0.08% *w*/*v*; adrenaline 1 mg/mL solution: 0.1% *v/v* in 0.9% saline) 10 min before liposuction. Around 30 mL of lipoaspirate was collected from each donor’s abdominal area with a cannula of 11 G, 2 holes, and a 20 mL VAC-Lock syringe. The fat was transported in an adiabatic container to the laboratory and processed within 24 h from harvest.

### 2.2. Production and Purification of Vibrio alginolyticus Collagenase

To obtain the *Vibrio alginolyticus*-based collagenase, the methodology reported by Di Pasquale et al. (2019) [44] was followed. Briefly, the *V. alginolyticus* strain was cultured at 30 °C and 150 rpm in a medium of peptone of animal origin and salts dissolved in purified water until the optical density at 60 nm (OD600) reached a value of 0.6.

Next, the inoculum was transferred into a fermenter to collect the collagenase secreted in the culture medium. Collagenase activity was determined spectrophotometrically using the modified Wunsch–Heidrich method [58]. Once the enzymatic activity reached more than 25,000 nkat/L, the fermentation was terminated and the temperature was lowered to 8 °C at 60 rpm for approximately 20 min. The fermented culture medium was clarified and concentrated by ultrafiltration. Then, the concentrated solution was dialyzed against 10 mM CaCl_2_, 25 mM TRIS–HCl, and pH 7.1 buffer. The solution from the previous step underwent a first purification by weak anion-exchange chromatography using a column prefilled with DE-52 resin (DEAE: diethylaminoethyl cellulose, Whatman, Maidstone, UK).

Collagenase activity was recovered within 3 to 5 bed-column volumes eluted with 700 mM NaCl, 10 mM CaCl_2_, 300 mM TRIS–HCl, and pH 7.1 buffer. The raw enzyme then underwent strong anion-exchange chromatography using a column pre-filled with Source 15Q resin (GE Healthcare, Chicago, IL, USA). The collagenase was eluted from the column with 10 mM CaCl_2_, 250 mM Tris–HCl, and pH 7.1 buffer. All chromatography runs were monitored using a UV–vis detector (GE Healthcare) at 280 nm.

Pooled fractions exhibiting collagenolytic activity underwent ultrafiltration using the Cogent system (Millipore, Burlington, MA, USA) equipped with 10 kDa cut-off modified PES membranes (Millipore) against 10 mM CaCl_2_, 25 mM TRIS–HCl, and pH 7.1 buffer. Routinely, a purified *V. alginolyticus* collagenase preparation from an 8-L solution exhibits 700 nKat/mL enzymatic activity.

### 2.3. Adipose Tissue Enzymatic Digestion

The study was divided into two main parts. The first part consisted of the optimization of the adipose digestion using a novel *Vibrio alginolyticus*-derived collagenase. For this purpose, the standard enzymatic digestion method consisting of 1 mg/mL of enzyme and 45 min of incubation time was selected as the control. Additionally, to optimize the *V. alginolyticus* collagenase process, the concentration of the enzyme and the incubation time were modified. The evaluated enzyme concentrations are displayed in Table 1 with the different evaluated incubation times for each concentration.

The lipoaspirate sample was divided into portions of 5 mL each for every evaluated combination of parameters. Every part was added into 5 mL of 1X Phosphate-Buffered Saline (PBS) with the evaluated concentration of *V. alginolyticus* collagenase and 2% Bovine Serum Albumin (BSA) and placed in agitation at 37 °C to evaluate different incubation times. Once the time passed, the digestion process was stopped with a complete growth medium (DMEM supplemented with 10% Fetal Bovine Serum, 1% of 1:1 penicillin/streptomycin and 0.6% Amphotericin B) and centrifuged for 5 min at 3000 rpm.

The obtained pellet of each portion was resuspended in 1 mL of complete growth media and filtered with a 70 µm cell strainer to be seeded in a T25 flask. The extracted cells were evaluated in terms of *cellular yield* and *clonogenic* and *proliferation capacity*, and the optimized parameters were further characterized with flow cytometry and differentiation potential.

The second part of the study consisted of the confrontation of the previously optimized method with two commercial *Clostridium histolyticum*-based collagenases: a *Clostridium histolyticum* blend and the standard enzyme used in the laboratory, Collagenase Type I (GIBCO life technology, USA). Both commercial collagenases were prepared in a concentration of 1 mg/mL in 1X Hank’s Balanced Salt Solution (HBSS) with 2% BSA. The adipose tissue was divided into portions of 5 mL and was enzymatically digested in 5 mL of each commercial collagenase solution at 37 °C for 45 min in agitation. The enzymatic action was blocked with a complete growth medium, and the following steps were executed as previously described. The extracted cells with the optimized method and the commercial enzymes were compared in terms of cellular yield, clonogenic and proliferation capacity, and cellular viability on expanded cells.

**Figure 1:**
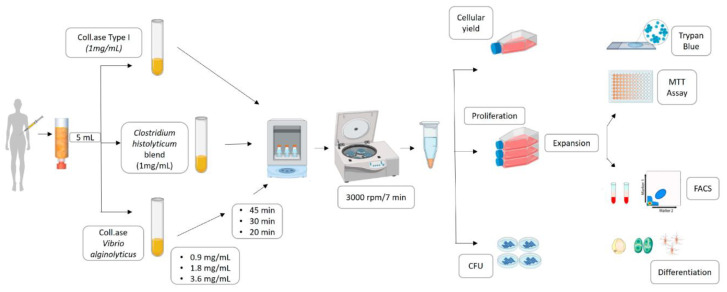
Experimental plan for the novel collagenase optimization method and comparison with commercial enzymes.

Intra- and inter-donor comparative analyses were performed. Figure 1 summarizes the followed methodology.

### 2.4. Cellular Yield

The extracted cells were counted in order to calculate the cellular yield, determined as the number of extracted free cells divided by the processed volume of fat. The number of living cells was calculated using the Trypan Blue exclusion assay in a CytoSMART counter (Automated Image-Based Cell Counter, version 1.5.0.16380, CytoSMART Technologies B.V., Eindhoven, The Netherlands).

### 2.5. Clonogenic Capacity

To evaluate the clonogenic capacity of the extracted cells, they were plated in triplicate in a 12-well plate at a concentration of 1000 cells/mL. The cells were incubated in a humidified atmosphere with 5% CO_2_ at 37 °C for 14 days, changing the medium every 48 h. The cells were stained with Toluidine Blue (Sigma-Aldrich, Milan, Italy) on the last study day to count the colonies. The colony former unit (CFU-F) was calculated as a percentage of the number of colonies divided by the number of seeded cells.

### 2.6. Proliferation Capacity

The extracted cells were seeded on a 25 cm^2^ T-flask with a complete culture medium and incubated in a humidified atmosphere at 37 °C with 5% CO_2_. The first medium change was performed after 72 h from the enzymatic digestion with subsequent changes every 48 h. The proliferation capacity was determined considering the required days to reach 80% confluence.

### 2.7. Immunophenotyping

Collected cells with the optimized enzymatic digestion immediately after Passage 0, along with subsequent subculture cells (Passage 4), were characterized by flow cytometry. The digested adipose tissue was centrifuged at 3000 rpm for 6 min. The cell pellet was incubated with 1 mL of erythrocyte lysis buffer 1X (Macs Miltenyi Biotec, Milan, Italy) for 10 min and filtered through a 70 µm cell strainer. Subsequently, cells were washed with 1 mL in PBS (1X) and incubated (1 × 10^5^ for each tube) with conjugated antibodies on ice for 30 min. After incubation, the pellets were centrifuged (5000 rpm, 7 min) and resuspended in 100 µL of PBS (1X).

The antibodies used were: CD45 FITC conjugate (1:20 dilution), CD34 PE conjugate (1:5 dilution), CD90 PE conjugate (1:20 dilution), CD73 BV421 conjugate (1:5 dilution), CD34 APC conjugate (1:20 dilution), CD146 APC conjugate (1:5 dilution), CD105 PE conjugate (1:5 dilution), and SEEA3 FITC conjugate (1:5). For cell viability, propidium iodide was used. All antibodies were purchased from BD Biosciences (Becton Dickinson Italy S.P.A., Milano, Italy). Immunophenotyping was performed through a chant II FACS (BD, Becton Dickinson, Milano, Italy).

### 2.8. Differentiation Assay

To evaluate the differentiation potential of the extracted cells with the optimized parameters, cells from passage 4 (P4) were used for both the treated and control groups. Stained cells were compared with differentiated cells extracted with Collagenase Type I, performed in triplicate. For adipogenic and osteogenic differentiation, 5000 cells were seeded in a 12-well plate in triplicate and incubated at 37 °C with 5% CO_2_. After 24 h of cell incubation, the complete culture medium was replaced with adipogenic and osteogenic mediums, respectively (StemPro osteogenesis differentiation Kit–GIBCO Life Technology, Monza, Italy). To determine adipogenic differentiation capability, cells were fixed after 14 days of study with Baker’s fixative (Bio-Optica, Milan, Italy) for 10 min at 4 °C as recommended by the manufacturer, washed for 10 min with tap water, and stained with Oil-Red-O ready-to-use solution (Bio-Optica, Milan, Italy) for 15 min and Mayer’s Hematoxylin (Bio-Optica, Milan, Italy) for 5 min. Finally, the cells were washed with tap water for 5 min and mounted with Mount Quick aqueous solution (Bio-Optica, Milan, Italy).

After 14 days of incubation, the osteogenic differentiation capacity was evaluated by staining the cells with Alzarin Red solution (Merck KGaA, Darmstadt, Germany) for 3 min post-fixation with 4% formaldehyde (Bio-Optica, Milan, Italy) in 0.05 M PBS for 30 min at 4 °C. The Alzarin Red was washed with distilled water and followed by immersion of the samples in Mayer’s Hematoxylin for 30 s. Finally, the glass coverslips were dehydrated in an ethanol gradient concluding with two passages in xylene to be mounted with Entellan (Merck KGaA, Darmstadt, Germany).

The differentiation in chondrogenic lineage was evaluated by resuspending 1 × 10^6^ cells in 5 µL of complete culture medium in triplicate and incubating for 2 h in a 12-well plate. After the time had passed, chondrogenic medium (StemPro chondrogenic differentiation Kit, GIBCO Life Technology, Monza, Italy) was added and changed every 3 days. After 21 days of study, the cells were fixed with 4% formaldehyde in PBS 0.05 M for 30 min at 4 °C. The fixative was washed with distilled water and the cells were stained with Alcian Blue solution (Merck KGaA, Darmstadt, Germany) for 40 min in slow agitation and later with Nuclear Fast Red (Bioptica, Milan, Italy) for 20 min. The samples were dehydrated and mounted with Entellan.

The same 4th passage cells with complete culture media were used as controls, and they were seeded and stained following the same procedure for every lineage. Once the samples were completely dried, the cells were imaged in light microscopy using an Olympus BX-51 microscope (Olympus, Tokyo, Japan) equipped with a digital camera (DKY-F58 CCD JVC, Yokohama, Japan) and connected to a PC endowed with Image-Pro Plus 7.0 software. The mounted samples were gently cleaned with ethanol and then placed on the microscope slide holder. A total of 5 images for each slide were acquired using a 20× objective for the quantification of lipid droplets, 10× objective for osteogenic quantification, and 4× for chondrogenic quantification. For each quantification, Photoshop software (Adobe Photoshop CS6 v13.0 extended) was used to isolate the specific differentiation staining color (red for lipid droplets and calcium deposits, blue for the cartilage-like matrix). Successively, a custom-designed ImageJ plugin (U.S. National Institutes of Health), in the blind condition was used to make a binary image and quantify the differentiation-specific color previously isolated. For adipogenic differentiation, the number of lipid droplets (referred to as a red spot on the cell cytoplasm) was quantified. For chondrogenic differentiation, the area of the chondrogenic aggregates (marked by Alcian blue staining) was considered. For osteogenic differentiation, the calcium deposit area (marked from the Alzarin Red staining) was quantified. After semi-quantification with ImageJ, the data were transferred into a PRISM file for statistical analysis and graph creation.

### 2.9. Cellular Viability Test

To evaluate the potential cytotoxicity activity of the *V. alginolyticus* collagenase in the optimized protocol, two viability tests were performed to compare it with Collagenase Type I and the *C. histolyticum* blend. The first viability test was the Trypan Blue exclusion test. Expanded cells (P4) were detached and divided into three portions, one for each evaluated collagenase. The viability of each part was measured with Trypan Blue solution in a CytoSMART counter (Automated Image-Based Cell Counter, version 1.5.0.16380, CytoSMART Technologies B.V., Eindhoven, Netherlands) and named viability pre-treatment. The cells were then placed in contact with the evaluated collagenases at the optimized concentration for *V. alginolyticus* collagenase and 1 mg/mL for Collagenase Type I and the *C. histolyticum* blend and incubated at 37 °C for 20 min in agitation. At the end of the incubation time, the viability post-treatment was measured with Trypan Blue, as previously mentioned. The test was performed in triplicate.

The second viability test was the methyl-thiazolyl-tetrazolium (MTT) colorimetric assay for metabolic activity. P4 cells were detached and divided into four portions, one with no treatment as the control. The remaining three were treated with the the evaluated enzymes at the optimized concentration for *V. alginolyticus* collagenase and 1 mg/mL for Collagenase Type I and the *C. histolyticum* blend. All four portions were incubated at 37 °C for 20 min in agitation. After the time had passed, 1 × 10^4^ cells were seeded in sextuplicate for each evaluated enzyme with 100 µL of complete growth medium in a 96-well plate. The cells were incubated for 24 h at 37 °C and 5% CO_2_. The medium was removed after the incubation time and the cells were washed with 1X PBS. The cells were treated with 100 µL of MTT (3-(4,5-dimethylthiazol-2-yl)2,5-diphenyltetrazolium bromide) at a concentration of 5 mg/mL (Sigma, Italy) and incubated for 4 h in the dark. Subsequently, the formed formazan crystals were dissolved in 100 µL of dimethyl sulfoxide (DMSO) for 10 min. The optical density was measured in a microplate reader (HTX Microplate Reader BioTek Instruments, Winooski, VT, USA) at a wavelength of 530 nm. The cell viability was calculated using Equation (1) as a percentage of the absorbance of the treated cells in relation to the absorbance of control cells. All measurements were performed in triplicate for each treatment and the control cells.

(1) $$\%cellsviability=\left(\frac{A_{Treatment}}{A_{Control}}\right)*100$$

Finally, the expanded cells treated with the evaluated enzymes were morphologically analyzed through a Transmission Electron Microscope (TEM) to evaluate cell integrity. The expanded cell pellets obtained after enzymatic treatments were fixed for 1 h in 2% glutaraldehyde in 0.1 M phosphate buffer solution (PBS) and, after washing, postfixed for 1 h in 1% OsO_4_ diluted in 0.2 M K_3_Fe (CN)_6_. After rinsing in 0.1 M PBS, the samples were dehydrated in graded concentrations of acetone and embedded in a mixture of Epon and Araldite (Electron Microscopic Sciences, Fort Washington, PA, USA). Ultrathin sections were cut at 70 nm thickness on an Ultracut E ultramicrotome (Reichert-Jung, Heidelberg, Germany), placed on Cu/Rh grids, and contrasted with lead citrate. Samples were observed with a Philips Morgagni 268 D electron microscope (Fei Company, Eindhoven, The Netherlands) equipped with a Megaview II camera for the acquisition of digital images.

### 2.10. Statistical Analyses

Statistical analyses were performed using GraphPad Prism 7.03 for Windows (GraphPad Software, La Jolla, CA, USA). The data are reported as means ± standard errors obtained after analyzing four consecutive patients. One-way analysis of variance (ANOVA) and the multiple comparisons test (Tukey test) were employed. A confidence interval of 95% was used to compare the evaluated experimental groups and a *p*-value < 0.05 indicated that the differences were statistically significant.

## 3. Results

### 3.1. Optimization of the Collagenase Fidia Adipose Tissue Digestion Process

#### 3.1.1. Cellular Yield, Clonogenic Potential, and Proliferation Capacity

**Figure 2:**
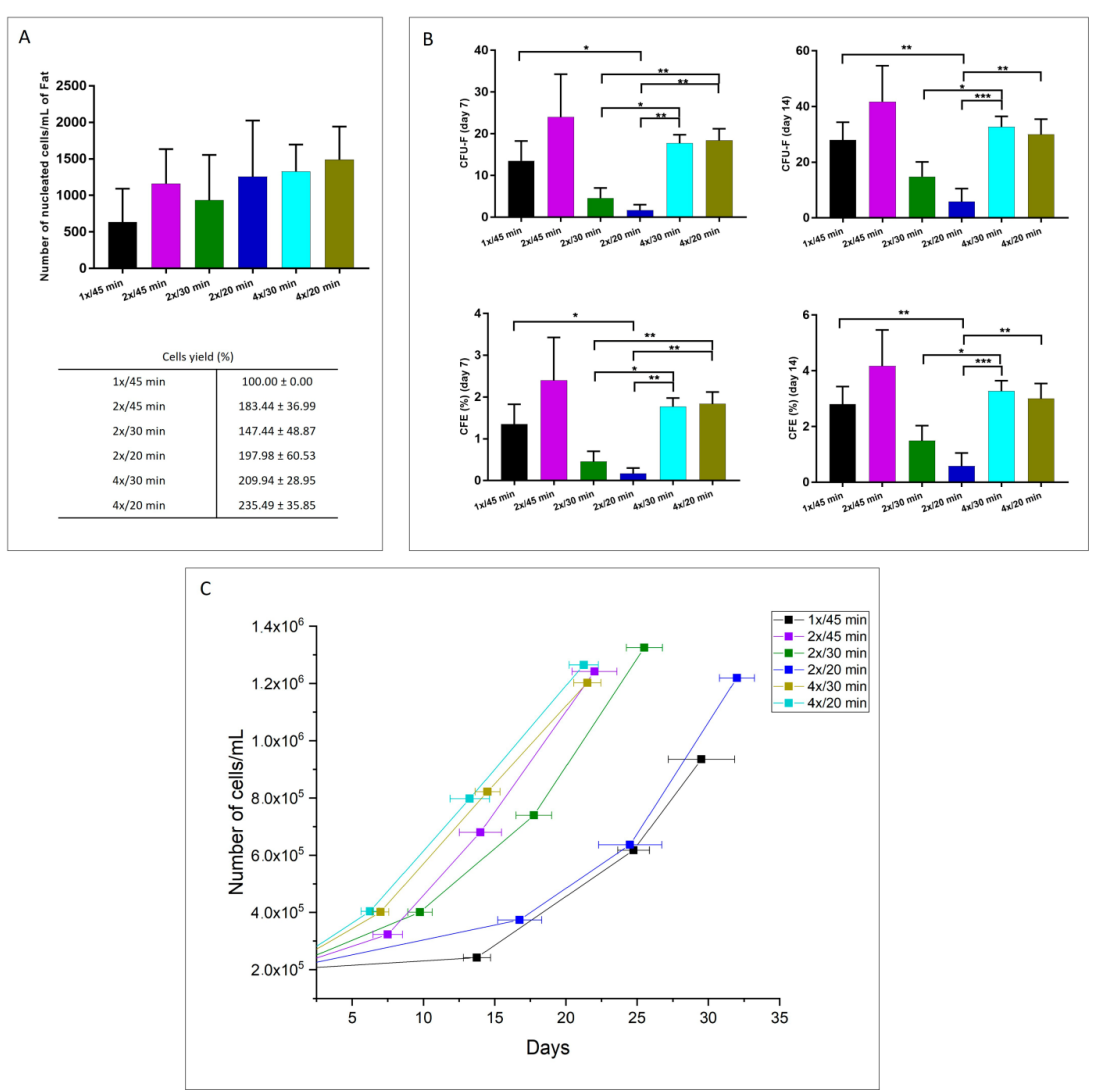
(**A**) Cellular yield, (**B**) clonogenic potential, and (**C**) cellular growth of extracted cells after enzymatic digestion with *V. alginolyticus* collagenase in varying concentrations and with different incubation times. The results are shown as means ± standard errors, indicating the significant statistical differences (*: *p*-value < 0.05, **: *p* ≤ 0.01, ***: *p* ≤ 0.001).

The extracted cells obtained after enzymatic digestion with *V. alginolyticus* collagenase in different concentrations and with different incubation times were analyzed for cellular yield, viability, proliferation capacity, and clonogenic potential. Figure 2A shows that the number of nucleated cells per mL of fat for all of the evaluated protocols was higher with the 4×/20 min treatment compared to the other protocols. Considering the enzymatic reaction at 1 mg/mL with an incubation time of 45 min as the “standard method” (cell yield 100%), the cellular yield of the 4×/20 min method resulted in a result of 235.49 ± 35.85%. However, there are no significant differences among the treatments. Figure 2B shows the relative proportion of CFU-F (colony-forming unit-fibroblast) evaluated 7 and 14 days after the seeding. The graph shows that the 2×/45 min protocol presents the highest among the treatments for extracting colony-forming units from adipose tissue. However, it can be seen from Figure 2C that cells extracted with the 4×/20 min protocol present a faster population doubling time, which means that these cells required fewer days to reach confluence in comparison with the other methods.

**Figure 3:**
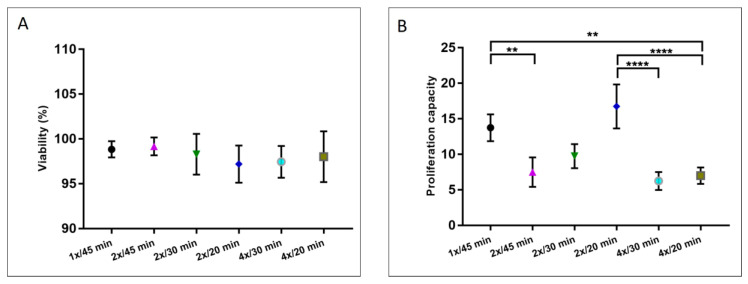
(**A**) Viability and (**B**) proliferation capacity of extracted cells after enzymatic digestion with *V. alginolyticus* collagenase in varying concentrations and with different incubation times. The results are shown as means ± standard errors, indicating the significant statistical differences (**: *p* ≤ 0.01, ****: *p* ≤ 0.0001).

Figure 3A shows the percentage of cellular viability obtained with the studied methods. As seen, there is no significant difference among the treatments in terms of cell viability, and for all the protocols, it was found to be over 96%. Additionally, Figure 3B presents the proliferation capacity of the different evaluated protocols. From the graph, the elevated proliferative function of the extracted cells is notorious after the 2×/20 min methodology compared to the other procedures.

Considering the obtained results, the selected optimized protocol was with a concentration of 4x and an incubation time of 20 min. The protocol was preferred given that its biological behavior is comparable with the other protocols, but it requires less time to digest adipose tissue.

#### 3.1.2. Immunophenotyping of Optimized Fidia Collagenase Method

**Figure 4:**
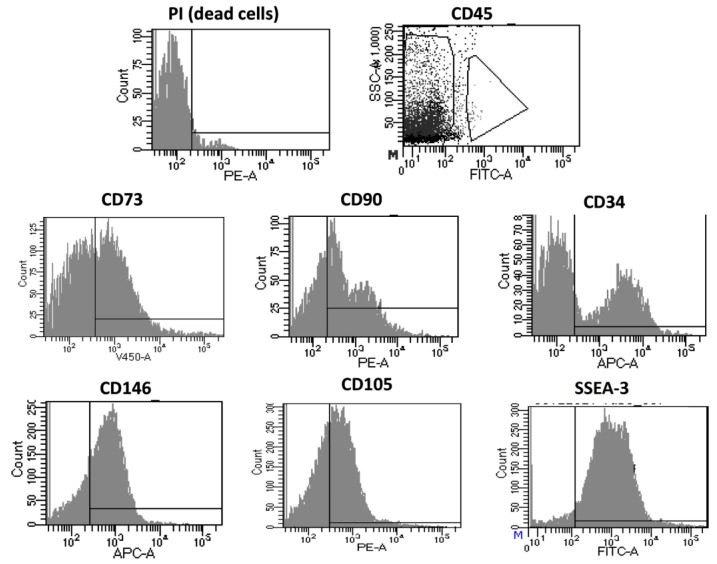
Immunophenotyping of the extracted cells with the optimized protocol of *V. alginolyticus* collagenase after adipose tissue enzymatic digestion (P0).

The cells extracted with the optimized protocol were characterized to identify the cellular composition of the suspension after the extraction (passage 0; P0) and after culturing them until passage 4 (P4). Figure 4 shows the FACS results at P0 for the different analyzed antibodies. It can be seen by the large cloud of data located on the negative side of the Propidium Iodide (PI) marker that most of the evaluated cells (approximately 99.8%) were alive, indicating that the results for the different antigens accurately represent the cellular population on the sample. Firstly, out of the cells evaluated for CD45, 6.4% were positive, representing a population of leucocytes. The remaining cells were negative for this marker which, alongside the positive expression of CD90, CD73, CD34, and CD105, characterize mesenchymal stem cells and cells of hematopoietic origin. In the same CD45-negative population, the positive expression of CD146 represents pericytes on the cellular suspension.

Additionally, the presence of another subpopulation, multilineage-differentiating stress-enduring cells (MUSE), was characterized by the simultaneous expression of CD105 and SEEA3.

**Figure 5:**
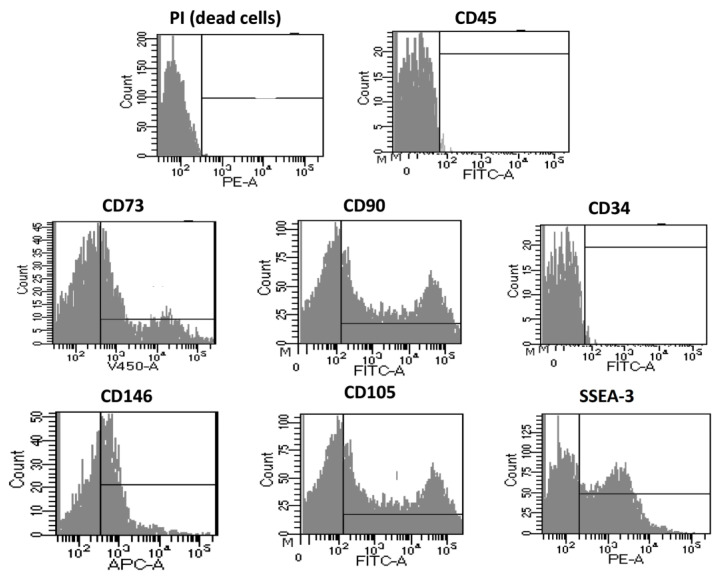
Immunophenotyping of extracted cells with the optimized protocol after the culture procedure until the cells reached passage 4 from the adipose digestion.

On the other hand, Figure 5 shows the cellular characterization of the extracted cells with the optimized protocol after being cultured until passage four. As seen with the P0 analysis, most of the evaluated cells were alive during the study. It can be seen that the cell expressed the typical markers for mesenchymal stem cells with a reduced presence of other subpopulations, as seen with the almost non-existent positive result for CD45 and CD34, markers for leucocytes and hematopoietic stem cells, respectively. Moreover, the cells maintain their multipotency, as proven by the simultaneous positive responses for the CD105 and SEEA3 markers.

#### 3.1.3. Analysis of Multipotency

**Figure 6:**
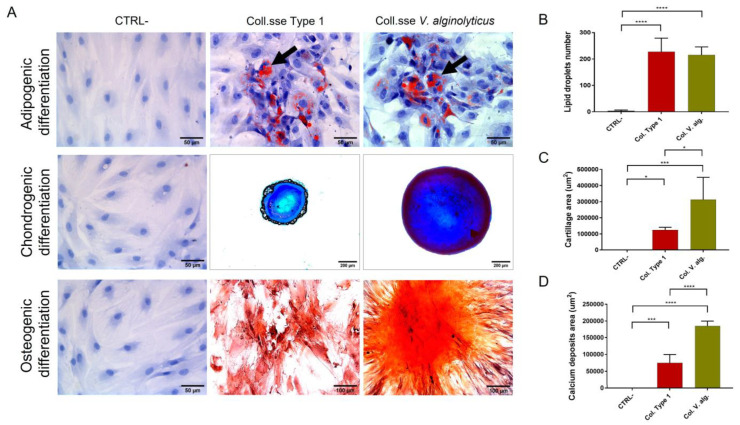
(**A**) Optical microscopy images of extracted cells with the *V. alginolyticus* optimized protocol compared with extracted cells with Col. Type I after being induced with differentiation medium (adipocytes present lipid droplets identified with black arrows, chondrocytes in blue, and osteocytes in red) in comparison with uninduced cells (CTRL-). Statistical graphs of semi-quantitative analysis of (**B**) lipid droplet number for adipogenic differentiation, (**C**) the cartilage-like matrix area for chondrogenic differentiation, and (**D**) calcium deposit areas for osteogenic differentiation. The results are shown as means ± standard errors, indicating the significant statistical differences (* *p* ≤ 0.05, *** *p* ≤ 0.001, **** *p* ≤ 0.0001).

**Figure 7:**
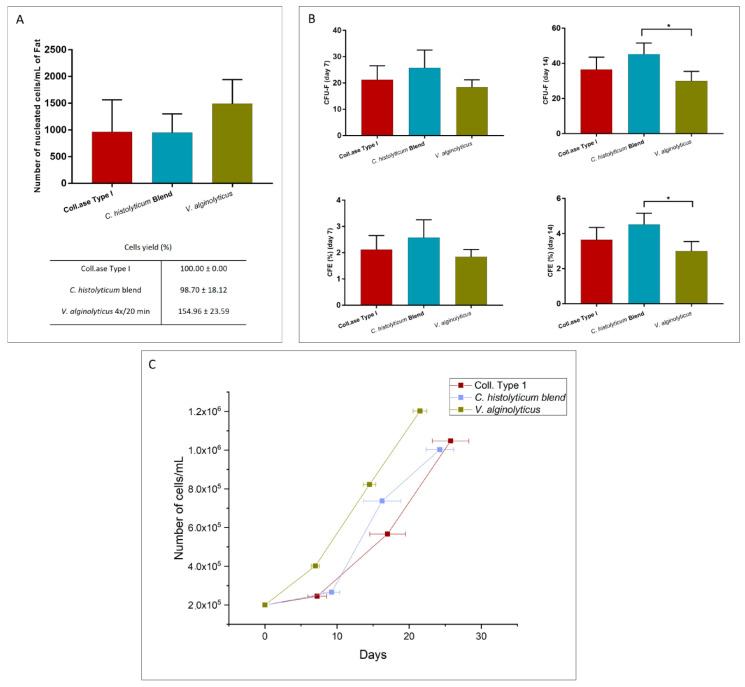
(**A**) Cellular yield, (**B**) clonogenic potential, and (**C**) cellular growth of extracted cells after enzymatic digestion with the optimized *V. alginolyticus* collagenase compared with Collagenase Type I and the *C. histolyticum* blend. The results are shown as means ± standard errors, indicating the significant statistical differences (*: *p*-value < 0.05).

The extracted cells show a differentiative capacity in the three evaluated cellular lineages after being seeded in the differentiative culture medium in the evaluated time. Figure 6A shows representative images of the differentiation process compared with the differentiated cells extracted with Collagenase Type I and the control (cells cultured in a complete growth medium). The semi-quantification analysis of lipid droplets presented in Figure 7B showed comparable adipogenic differentiation between *V. alginolyticus* collagenase (215.3 ± 17.5, n = 3) and Collagenase Type I (227.1 ± 29.7, n = 3), while the negative control resulted in 3.867 ± 1.348, n = 3. Otherwise, *V. alginolyticus* collagenase resulted in a statistically significant higher differentiation potential for chondrogenic (313.114 µm^2^ ± 79.755 µm^2^, n = 3) and osteogenic (185.119 µm^2^ ± 8.431 µm^2^, n = 3) lineages when compared to Collagenase Type I (124.643 µm^2^ ± 8.240 µm^2^, n = 375.572 µm^2^ ± 13.652 µm^2^, n = 3) and negative controls (0 ± 0, n = 3, 31.73 ± 18.67, n = 3), as seen in Figure 7C and Figure 7D, respectively. The results showed that the extracted cells with the optimized treatment present the capacity to proliferate in adipocytes, osteocytes, and chondrocytes.

### 3.2. Comparison of the Optimized Protocol with Commercial Collagenases

#### 3.2.1. Cellular Yield, Clonogenic Potential, and Proliferation Capacity

Figure 7A shows the number of cells obtained after the three enzymatic treatments in the study. As seen in the graphic, an average of 1.49 × 10^3^ ± 2.27 × 10^2^ cells is obtained with *V. alginolyticus* collagenase at 4x/20 min, while in the case of the two control treatments, the average cell numbers were 9.64 × 10^2^ ± 3 × 10^2^ and 9.51 × 10^2^ ± 1.75 × 10^2^ for Collagenase Type I and the *C. histolyticum* blend, respectively. Considering Collagenase type I as the “gold standard”, and therefore with a cellular yield of 100% in terms of the number of extracted cells per mL of processed adipose tissue, the cellular yield percentage of the *V. alginolyticus* collagenase at 4×/20 min was 154.96 ± 23.59, proving it to be more efficient, whereas for the *C. histolyticum* blend, the cellular yield percentage was of 98.70 ± 18.12. However, there was no statistical significance among the data. Figure 7B reports the number of Fibroblast-like Colony Forming Units (CFU-F) for the evaluated enzymatic treatments counted 7 and 14 days after enzymatic digestion. The average CFU-F obtained with the *V. alginolyticus* collagenase at 4×/20 min were 18.42 ± 1.40 and 30 ± 2.74 after 7 and 14 days, respectively. In the case of both control collagenases, the average numbers of CFU-F after 7 days were 21.25 ± 2.66 and 25.75 ± 3.40 for Collagenase Type I and the *C. histolyticum* blend, respectively. Even though the number of CFU-F for the *V. alginolyticus* collagenase after 7 days is lower than the other two treatments, there is no statistical significance among the data. Notwithstanding, after 14 days, the number of CFU-F for the *C. histolyticum* blend (45.25 ± 3.20) is statistically higher than the one reported for the *V. alginolyticus* collagenase, but there was no difference with the average number for Collagenase Type I (36.50 ± 3.52). The graph also shows the clonogenic efficiency (CFE) at 7 and 14 days. The *V. alginolyticus* collagenase at 4×/20 min reports a CFE lower than the *C. histolyticum* blend at 7 days and the CFE was even lower after 14 days.

Figure 7C shows the cell growth curve of the evaluated enzymatic treatments. To assess the proliferative capacity of each product, after the enzymatic digestion, 2 × 10^5^ cells were seeded for every treatment, and the time and number of cells were evaluated once the plates reached confluence. The proliferative capacity is comparable between the three methods with no statistical differences.

**Figure 8:**
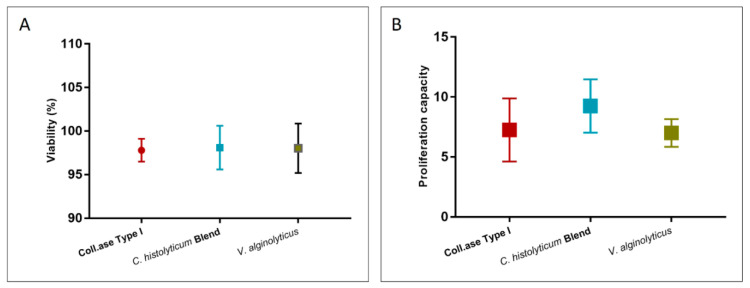
(**A**) Viability and (**B**) proliferation capacity of extracted cells after enzymatic digestion with the optimized *V. alginolyticus* collagenase compared with Collagenase Type I and the *C. histolyticum* blend. There are no statistical differences among the data.

Figure 8 shows the cellular viability and proliferation capacity of the products extracted with the three enzymatic methods. It can be seen from the figure that there is no significant difference among the treatments. In terms of cell viability, all the evaluated methods present a viability percentage over 95%, while the proliferative capacity is over 5 days to confluence.

#### 3.2.2. Cellular Integrity Assay

To evaluate whether the studied collagenases preserve the integrity of the cell membrane, expanded ASCs were subjected to treatment with the three enzymes being assessed (*V. alginolyticus* collagenase 4×/20 min, Collagenase Type I, and a *C. histolyticum* blend) for 20 min.

**Figure 9:**
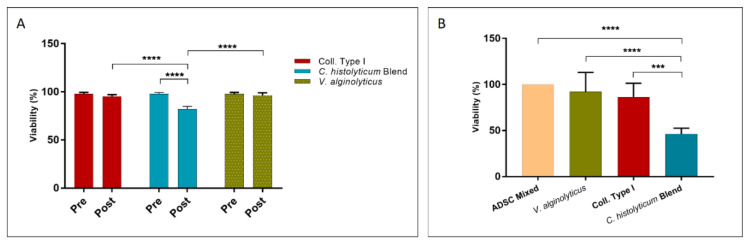
Viability percentage of expanded cells placed in contact with the optimized *V. alginolyticus* collagenase compared with Collagenase Type I and the *C. histolyticum* blend for 20 min evaluated with the (**A**) trypan blue exclusion test and (**B**) MTT test. The results are shown as means ± standard errors, indicating the significant statistical differences (***: *p* ≤ 0.001, ****: *p* ≤ 0.0001).

Cellular viability was measured with the Trypan Blue mortality test before (pre) and after (post) treatment with the collagenases. Figure 9A shows the mean percentage value of viability, where it is noticeable that there is no difference between pre- and post-treatment for *V. alginolyticus* collagenase 4×/20 min and Collagenase Type I, but a decrease in viability for cells treated with the *C. histolyticum* blend is appreciable (pre-viability 97.81 ± 0.92%, post-viability 82.03 ± 1.69%).

Figure 9B shows the viability percentage of cells treated with the evaluated enzymes after 24 h of treatment. This was assessed with the MTT test and compared with control cells (cells not subjected to enzyme exposure). The cells treated with *V. alginolyticus* collagenase 4×/20 min and Collagenase Type I present viability of 94.88 ± 17.88% and 86.46 ± 6.06%, respectively. The *C. histolyticum* blend, on the other hand, shows a viability percentage of 46.16 ± 2.59%, which is nearly half of that for *V. alginolyticus* collagenase.

**Figure 10:**
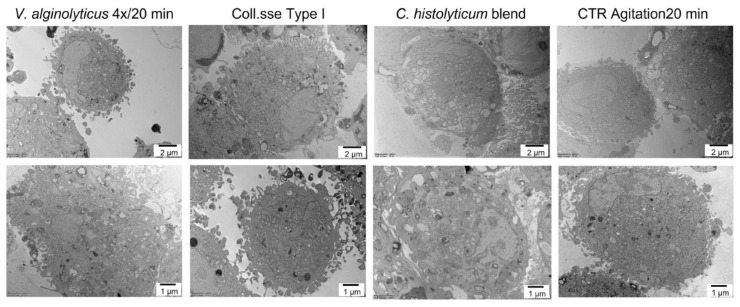
Representative images of TEM analysis of P4 cells treated with the optimized *V. alginolyticus* collagenase compared with Collagenase Type I and the *C. histolyticum* blend for 20 min. Cells placed in agitation for 20 min acted as the control group.

Finally, the cells were morphologically evaluated through transmission electron microscopy. Figure 10 shows some TEM-selected images of P4 cells treated with the evaluated collagenases. The cells treated with the *V. alginolyticus* collagenase present a regular profile with characteristics of cellular activation and polarization; in fact, many cytoplasmic membrane extroversions are noted. The same aspects can be seen in the cells treated with Collagenase Type I. Both treatments allow the release of extracellular vesicles following the formation of sessile and pedunculated bubbles on the cytoplasmic membrane. On the other hand, the cells treated with the *C. histolyticum* blend collagenase show irregularities in shape and size, with most of the cells characterized by diffuse cytoplasmatic vacuolization and autophagosomes, which indicate cellular degenerative processes.

Finally, the control cells present a rounded morphology consistent with the literature. The cytoplasm is well conserved with smooth endoplasmic reticulum activation, and the plasmalemma shows short microvillar protrusions.

## 4. Discussion

Non-specific proteolytic enzymes are extensively used in clinical practice. There are many diseases caused by uncontrolled collagen accumulation, such as fibrous skin and scars, or due to the production of necrotic connective tissues such as in chronic ulcers or burns. In these cases, the use of specific proteolytic enzymes represents an excellent therapeutic strategy [59,60,61]. Furthermore, the clinical use of collagenase in relation to adipose tissue has been used indirectly for cellulite, whose treatment targets adipose tissue, dermis, and fibrous septae with varying degrees of success and duration of response [62,63].

Bacterial collagenases are a usual proteolytic family of enzymes responsible for the disintegration of tissue. Among these, the most recognized in medical practice is the lyophilized extract of *Clostridium histolyticum* [64]. However, collagenases extracted from *C. histolyticum* possess reduced protein selectivity, degrading not only collagen but other essential proteins as well [44], possibly influencing product regenerative capacity. Although *C. histolyticum* collagenase has been used for over four decades in the laboratory, it is not suited for clinical practice given its long-lasting and non-standardized procedure [29,65]. The traditional method for isolation and culture of primary ASCs from adipose tissue relies on enzymatic digestion with collagenase, followed by multiple steps of centrifugation [66]. It is remarkable that there is no standardized protocol to isolate ASCs for clinical application. Notwithstanding, in Europe, these protocols have to fall within the legislative directives set by the European Medicines Agency (EMA), particularly the enzymatic digestion of a tissue to release cells as this is considered to be substantial manipulation [65]. If the enzymatic digestion causes the isolation of functionally intact tissue units or there is scientific evidence that the original structural and functional characteristics are maintained, the procedure is not considered substantial manipulation.

Collagenases extracted from *Vibrio alginolyticus* are primarily unexplored and understudied. Nevertheless, their uses are different and of extreme effectiveness in the wound fields [44,50,52]. Recently, its use has also been expanded to Dupuytren’s disease, after the withdrawal of *Clostridium hystolyticum* collagenase from the European Union for commercial reasons [67,68]. Animal model studies have demonstrated that collagenase from *Vibrio alginolyticus* does not cause skin necrosis, and no skin tears or wound dehiscences were observed, demonstrating the safety of this novel collagenase. This macroscopic data was confirmed by microscopic analysis, where no hematomas were found around the fibrotic area with the absence of leukocyte infiltrates and macrophages, confirming the selectivity for collagens I and III and reducing the risk of vascular lesions or skin ulcerations [41]. This manuscript aimed to clarify the effects of a novel collagenase blend from the *V. alginolyticus* strain and compare it with standard collagenases with regard to cell isolation, cellular yield, cell viability, and number of extracted living cells from human adipose tissue.

The novel *V. alginolyticus* collagenase was evaluated at different concentrations and with different periods of incubation. It was found that all evaluated protocols presented no statistical differences with respect to the number of extracted cells, cellular viability, and cellular growth. However, regarding cellular growth, the mean necessary days to confluence were lower for the concentration of 3.6 mg/mL, with incubation times of 30 and 20 min, than the rest of the protocols, which indicates a faster replication capacity that might influence cellular behavior in vivo. Even though both protocols presented similar results for all the evaluated parameters, the one with the faster enzymatic reaction is preferable. The optimized method based on a concentration of 3.6 mg/mL of *V. alginolyticus* collagenase and 20 min of incubation was used for further characterization. The FACS data showed that the extracted cells presented with stem cell characteristics, preserving the distinctive phenotype. Additionally, these cells are able to differentiate in three mesodermal cellular lineages (adipocytes, chondrocytes, osteocytes) and are comparable with the extracted cells after the Collagenase Type I enzymatic reaction. The higher chondrogenic and osteogenic potential shown by the *Vibrio* collagenase in the multipotency analysis could be due to the higher integrity of extracellular and membrane proteins and cellular receptors, essential for signaling and differentiation processes.

Additionally, the optimized protocol was compared with the standard enzyme for laboratory procedures and a commercial *C. histolyticum*-based collagenase. It was found that, in terms of cellular yield, clonogenic potential, and proliferation capacity, there are no differences between the optimized *V. alginolyticus* collagenase and the standard ones. Even though there were no statistical differences among the data, the results showed that the mean value for the optimized protocol cellular yield was higher than those reported for both C. *histolyticum*-based collagenases, probably due to the fact that the *V. alginolyticus* collagenase presented selectivity for collagen. Furthermore, it was demonstrated with the cellular growth curve that the extracted cells with the *V. alginolyticus* collagenase reach confluence faster than the other two evaluated enzymes.

Finally, it was seen that the evaluated enzymes in direct contact with stem cells provoke different reactions affecting cellular vitality. It was found that cells in contact with the *C. histolyticum* blend enzyme presented a considerable vitality reduction after 45 min of treatment; meanwhile, cells in contact with the optimized method presented the highest vitality among the treatments.

These findings show that while the *V. alginolyticus* collagenase has a comparable enzymatic function to the *C. histolyticum*-based collagenases, it does not affect additional structures that might be of vital importance in tissue regeneration applications. The enzymatic characteristics of the *V. alginolyticus* collagenase are gentle on extracellular matrix structures as they are selective in their degradation capacity [46]. Providing a gentle enzymatic therapy for the remotion of damaged tissue in skin defect treatment may improve the efficacy of novel approaches for tissue regeneration [69].

## 5. Conclusions

Using the novel *Vibrio alginolyticus*-based collagenase at a concentration of 3.6 mg/mL and 20 min of incubation time at 37 °C, the highest enzymatic efficiency with the shortest incubation time was found in comparison with the other evaluated parameters. Additionally, the selected method showed comparable efficiency with two commercial collagenases, Collagenase Type I (GIBCO life technology) and a *C. histolyticum* blend, but without affecting cellular integrity when used on expanded cells.

The cells extracted with the *Vibrio alginolyticus* collagenase at a 3.6 mg/mL concentration presented the phenotypic characteristics of stem cells and can differentiate into three mesenchymal lineages, showing the potential applications of this enzyme in different areas of regenerative medicine.

## Figures and Tables

**Table 1:** Name code of the evaluated enzyme concentration of *V. alginolyticus*-based collagenase with the evaluated incubation time for each concentration.

Name Code	Enzyme Concentration (mg/mL)	Incubation Time (min)
1x	0.9	45
2x	1.8	45, 30, 20
4x	3.6	30, 20

## Data Availability

The datasets generated and/or analyzed during the current study are available from the corresponding author upon reasonable request.
